# Safety, infectivity and immunogenicity of a genetically attenuated blood-stage malaria vaccine

**DOI:** 10.1186/s12916-021-02150-x

**Published:** 2021-11-22

**Authors:** Rebecca Webster, Silvana Sekuloski, Anand Odedra, Stephen Woolley, Helen Jennings, Fiona Amante, Katharine R. Trenholme, Julie Healer, Alan F. Cowman, Emily M. Eriksson, Priyanka Sathe, Jocelyn Penington, Adam J. Blanch, Matthew W. A. Dixon, Leann Tilley, Michael F. Duffy, Alister Craig, Janet Storm, Jo-Anne Chan, Krystal Evans, Anthony T. Papenfuss, Louis Schofield, Paul Griffin, Bridget E. Barber, Dean Andrew, Michelle J. Boyle, Fabian de Labastida Rivera, Christian Engwerda, James S. McCarthy

**Affiliations:** 1 QIMR Berghofer Medical Research Institute, Brisbane, Australia; 2 Current address: PharmOut, 111 Eagle Street, Brisbane, Queensland 4000 Australia; 3 Liverpool School of Tropical Medicine, Liverpool, UK; 4 Centre of Defence Pathology, Royal Centre for Defence Medicine, Joint Hospital Group, Birmingham, UK; 5 The University of Queensland, Brisbane, Australia; 6 The Walter and Eliza Hall Institute of Medical Research, Melbourne, Australia; 7 Department of Microbiology and Immunology, University of Melbourne, Melbourne, Australia; 8 Department of Medical Biology, University of Melbourne, Melbourne, Australia; 9 Current address: Medicines Development for Global Health Limited, 18 Kavanagh Street, Southbank, Victoria 3006 Australia; 10 Bio21 Molecular Science and Biotechnology Institute, Melbourne, Australia; 11 Department of Biochemistry and Molecular Biology, University of Melbourne, Melbourne, Australia; 12 The Peter Doherty Institute for Infection and Immunity, Melbourne, Australia; 13 Department of Medicine, Royal Melbourne Hospital, Melbourne, Australia; 14 Burnet Institute, Melbourne, Australia; 15 Current address: GSK, 436 Johnston Street, Abbotsford, Victoria 3067 Australia; 16 Australian Institute of Tropical Health and Medicine, James Cook University, Cairns, Australia; 17 Department of Medicine and Infectious Diseases, Mater Hospital and Mater Research, Brisbane, Australia

**Keywords:** Malaria, Vaccine, *Plasmodium falciparum*, Genetically attenuated, KAHRP, PfEMP1

## Abstract

**Background:**

There is a clear need for novel approaches to malaria vaccine development. We aimed to develop a genetically attenuated blood-stage vaccine and test its safety, infectivity, and immunogenicity in healthy volunteers. Our approach was to target the gene encoding the knob-associated histidine-rich protein (KAHRP), which is responsible for the assembly of knob structures at the infected erythrocyte surface. Knobs are required for correct display of the polymorphic adhesion ligand *P. falciparum* erythrocyte membrane protein 1 (PfEMP1), a key virulence determinant encoded by a repertoire of *var* genes.

**Methods:**

The gene encoding KAHRP was deleted from *P. falciparum* 3D7 and a master cell bank was produced in accordance with Good Manufacturing Practice. Eight malaria naïve males were intravenously inoculated (day 0) with 1800 (2 subjects), 1.8 × 10^5^ (2 subjects), or 3 × 10^6^ viable parasites (4 subjects). Parasitemia was measured using qPCR; immunogenicity was determined using standard assays. Parasites were rescued into culture for in vitro analyses (genome sequencing, cytoadhesion assays, scanning electron microscopy, *var* gene expression).

**Results:**

None of the subjects who were administered with 1800 or 1.8 × 10^5^ parasites developed parasitemia; 3/4 subjects administered 3× 10^6^ parasites developed significant parasitemia, first detected on days 13, 18, and 22. One of these three subjects developed symptoms of malaria simultaneously with influenza B (day 17; 14,022 parasites/mL); one subject developed mild symptoms on day 28 (19,956 parasites/mL); and one subject remained asymptomatic up to day 35 (5046 parasites/mL). Parasitemia rapidly cleared with artemether/lumefantrine. Parasitemia induced a parasite-specific antibody and cell-mediated immune response. Parasites cultured ex vivo exhibited genotypic and phenotypic properties similar to inoculated parasites, although the *var* gene expression profile changed during growth in vivo.

**Conclusions:**

This study represents the first clinical investigation of a genetically attenuated blood-stage human malaria vaccine. A *P. falciparum* 3D7 *kahrp*– strain was tested in vivo and found to be immunogenic but can lead to patent parasitemia at high doses.

**Trial registration:**

Australian New Zealand Clinical Trials Registry (number: ACTRN12617000824369; date: 06 June 2017).

**Supplementary Information:**

The online version contains supplementary material available at 10.1186/s12916-021-02150-x.

## Background

There is currently a strong commitment to eradicate malaria in the 21st century. This ambitious goal will be facilitated by the development of an effective vaccine. The past 20 years have seen over 100 clinical trials of malaria vaccine candidates, with the vast majority testing sub-unit vaccines [1]. To date, only one vaccine (RTS,S/AS01) has demonstrated protective efficacy against clinical malaria in a phase 3 trial, although protection is only partial [2]. RTS,S/AS01 has been approved by the European Medicines Agency, and a pilot implementation program coordinated by the World Health Organization is underway in Africa [3]. Given the overall slow progress of current vaccine strategies, there is a clear need for novel approaches.

Live, whole cell vaccines offer several potential advantages over subunit vaccines against a range of pathogens including malaria parasites. These include exposure to a greater range of antigens that are delivered to the correct anatomical compartments for immunological priming, as well as providing the correct pattern recognition signals to the immune system, which are specific for the pathogen class [4]. Various whole sporozoite vaccines that target the pre-erythrocytic stage of malaria parasite development have demonstrated promising results in preclinical studies, human challenge studies, and in clinical trials in malaria-endemic areas [1]. Strategies for sporozoite attenuation have included the use of radiation [5] or targeted gene deletion [6–8] to produce genetically attenuated parasites (GAP). Naturally acquired immunity to malaria is complex, with evidence suggesting that repeated exposure to blood-stage infection is an important component and is associated with an antibody response against merozoites and infected erythrocytes [9]. However, in the modern era, only a single live whole parasite blood-stage vaccine candidate has been tested in humans, in a pilot study undertaken using chemically attenuated parasites [10]. We aimed to develop a blood-stage GAP malaria vaccine and characterize its safety, infectivity, and immunogenicity in a phase 1 clinical trial.

*P. falciparum* erythrocyte membrane protein 1 (PfEMP1) is a polymorphic adhesion ligand displayed on knob-like structures on the surface of *P. falciparum*-infected erythrocytes, with specific types binding a variety of host receptors present on the vascular endothelium [11]. This arrangement facilitates attachment and sequestration of the infected erythrocyte in the microvasculature, thereby protecting parasites from clearance by the spleen [12]. Further, parasite sequestration is a critical determinant of malaria pathogenesis as it leads to the accumulation of infected erythrocytes in vital organs [13]. PfEMP1 is also a target of protective immunity [14] and has been shown to have an immunomodulatory effect [15]. PfEMP1 is a variable surface antigen, encoded by a repertoire of around 60 *var* genes per genome [16]. Thus, creation of a genetically modified parasite lacking PfEMP1 would require a targeted genetic modification that would prevent transcription of any *var* gene allele, an approach that may be technically challenging. Further, if such a result were achieved it would result in no expression of PfEMP1, a protein which has been proposed to be the target of strain-specific immunity [14].

The in vivo virulence and immunogenicity of a knobless *P. falciparum* clone selected under in vitro culture was investigated in Aotus monkeys by Langreth and Peterson [17]. After multiple inoculations with the knobless parasites, non-splenectomized animals either did not develop patent parasitemia, or exhibited very low parasitemia, with infections self-resolving. In contrast, splenectomized animals developed significant infections when inoculated with knobless parasites. This study indicated the importance of the knobs on the surface of infected erythrocytes in enabling parasites to sequester to avoid splenic clearance. When monkeys with intact spleens were exposed to knobless parasites they developed a humoral immune response and exhibited partial protection when subsequently challenged with knob-positive parasites. Together, these data suggest a knob-minus live malaria vaccine represents an attractive approach.

Our approach to develop a blood-stage GAP vaccine was to target the gene encoding the knob-associated histidine-rich protein (KAHRP), which is responsible for the assembly of knob structures at the infected erythrocyte surface. In the absence of KAHRP, *P. falciparum*-infected erythrocytes lack knob-structures and cannot correctly display PfEMP1 [18]. Building on the early studies in *Aotus* monkeys, we hypothesized that the loss of KAHRP would result in a significant loss of fitness in vivo due to enhanced parasite clearance accompanied by priming of the immune system from lack of parasite sequestration and increased passage to the spleen. The production of the blood-stage GAP vaccine under Good Manufacturing Practice (GMP) by targeted deletion of the gene encoding KAHRP has been described previously [19]. Here we report the results of a clinical trial to characterize the safety, infectivity, and immunogenicity of the *P. falciparum kahrp*– blood-stage GAP vaccine in healthy volunteers.

## Methods

### Study design and participants

This phase 1 study was planned to be composed of two parts. The first part, performed as planned, was an open-label, dose-escalation study to determine the safety, infectivity, and immunogenicity of single doses of the GAP vaccine administered intravenously. The second part was planned to provide preliminary information on the efficacy of the GAP vaccine by determining the protective immune response of GAP vaccination to subsequent challenge with wild-type blood-stage *P. falciparum*. However, it was decided not to proceed with the second part due to the lack of complete attenuation of the GAP vaccine, and alloimmunization events, observed in the first part (see the “Results” section). The study design and methodology for the second part of the study are not described here.

Healthy malaria naïve males aged 18 to 55 years were eligible for inclusion in the study (for full eligibility criteria see Additional File 1: Text S1). The study was conducted at Q-Pharm (Brisbane, Australia) and was registered on the Australian and New Zealand Clinical Trials Registry (Trial ID: ACTRN12617000824369; date registered: 06 June 2017).

### GAP vaccine

The manufacture of the GAP vaccine has been described previously [19]. Briefly, genetically attenuated blood-stage malaria parasites were produced by targeted deletion of *kahrp* from *P. falciparum* strain 3D7, and parasites were cultured in a wave bioreactor under GMP-compliant conditions. The cryopreserved GAP master cell bank was tested for sterility, absence of viral contaminants and endotoxins, and parasite identity, viability, and antimalarial drug sensitivity. The GAP master cell bank has a parasitemia of 6.3% with 96% of parasites at ring stage. The GAP vaccine inoculum used for each subject in this study was prepared aseptically from a single vial of the GAP master cell bank by thawing, washing, and suspending the appropriate number of infected erythrocytes in 0.9% sodium chloride for injection in a final volume of 2 mL. Syringes containing the inocula were stored at a controlled temperature for a maximum of 2 h before administration.

### Procedures

The study was conducted in four consecutive dose cohorts of two subjects each. Sample size was not based on formal statistical calculations but was considered appropriate to achieve the study objectives. Following a screening period of up to 28 days, subjects were inoculated intravenously with the GAP vaccine (day 0). A sentinel dosing strategy was used for the first three cohorts whereby the first subject in each cohort was inoculated with the GAP vaccine at least 24 h before the second subject. Subjects in cohort 4 were inoculated with the GAP vaccine concurrently since no significant safety signals had been observed in the preceding cohorts.

Parasitemia was monitored throughout the study by collecting blood samples and performing quantitative PCR (qPCR) targeting the gene encoding *P. falciparum* 18S rRNA [20]. Subjects were to receive a standard curative course of artemether/lumefantrine treatment if parasitemia reached ≥5000 parasites/mL (≥200,000 parasites/mL for cohort 4) or if clinical symptoms of malaria were observed (clinical symptom score > 6), or on day 28±3 (day 60±3 for cohort 4) if the preceding treatment criteria had not been reached earlier. The end of the study visit occurred on day 90 for safety and immunogenicity assessments.

The GAP vaccine dose administered to cohort 1 was 1800 viable parasites which is the dose of *P. falciparum* 3D7 wild-type parasites routinely used in previous controlled human malaria infection (CHMI) studies using the induced blood-stage malaria (IBSM) model [21–23]. A formal Safety Review Team meeting was conducted between cohorts to assess the safety of the GAP vaccine and decide whether to dose escalate and proceed with the next cohort. The planned dose escalation was 1.8 × 10^5^ viable parasites for cohort 2 (100-fold increase from cohort 1) and 3 × 10^6^ viable parasites for cohort 3 (a dose of parasites equivalent to that released from the liver after a five mosquito bite CHMI study [24]). Based on the results observed in cohort 3 (with both subjects developing parasitemia but remaining asymptomatic), the study protocol was amended to include a fourth cohort administered the same GAP vaccine dose as cohort 3 (3 × 10^6^ viable parasites), but with the antimalarial treatment criteria amended with a longer follow-up to allow for better characterization of parasite infectivity. A planned protocol deviation was performed for cohort 3 due to the unexpected results observed; subject 6 was administered antimalarial treatment on day 35 when parasitemia reached 5000 parasites/mL instead of the protocol stipulated day 28. This was considered reasonable since the subject was asymptomatic and consented to the additional period of follow-up. The QIMR Berghofer Medical Research Institute Human Research Ethics Committee was notified of this decision.

### Safety assessments

Safety assessments were performed throughout the study and included adverse event (AE) recording, physical examinations, vital signs monitoring, electrocardiographs (ECGs), clinical laboratory evaluation (hematology, biochemistry, and urinalysis), and malaria clinical score recording [25]. The severity of AEs was graded according to a set of criteria developed for guidance of commonly reported symptoms, signs, and abnormal laboratory findings in malaria challenge studies (mild = grade 1; moderate = grade 2; severe = grade 3). These were adapted from the Common Terminology Criteria for Adverse Events v3.0 [26].

### Immunogenicity assessments

The development of an antibody response to the GAP vaccine was determined by collecting blood samples and using standard ELISA methods to measure IgG and IgM antibodies towards *P. falciparum* merozoite surface protein 2 (MSP2) and to whole parasite lysate (Additional File 2: Text S1) [27, 28]. The development of a cell-mediated immune response to the GAP vaccine (cohorts 1-3 only) was determined by collecting blood samples and using flow cytometry to identify T cell subsets (Additional File 2: Text S2, Table S1, and Figure S1) [29, 30].

### In vitro analysis of parasites rescued into culture

A series of in vitro analyses were performed on parasites isolated from the blood of subjects at maximum parasitemia prior to antimalarial treatment. Parasites were cultured directly from blood samples to reach a target parasitemia of 2-8% with > 50% ring stage parasites before cryopreservation. The properties of the isolated parasites were compared to parasites inoculated on day 0. Genotyping of the parasite genetic material was performed using standard molecular biology techniques including Sanger sequencing and whole genome sequencing (Additional File 3: Text S1) [31–36]. The cytoadherence phenotype of the isolates was determined using both static and flow adhesion assays (Additional File 3: Text S2) [37, 38]. Scanning electron microscopy was performed to characterize the knob status of parasite-infected erythrocytes (Additional File 3: Text S3) [39]. The *var* gene expression profile of parasites was determined using diagnostic RT-PCR (Additional File 3: Text S4 and Table S1) [40–43].

## Results

### Subjects

**Fig. 1:**
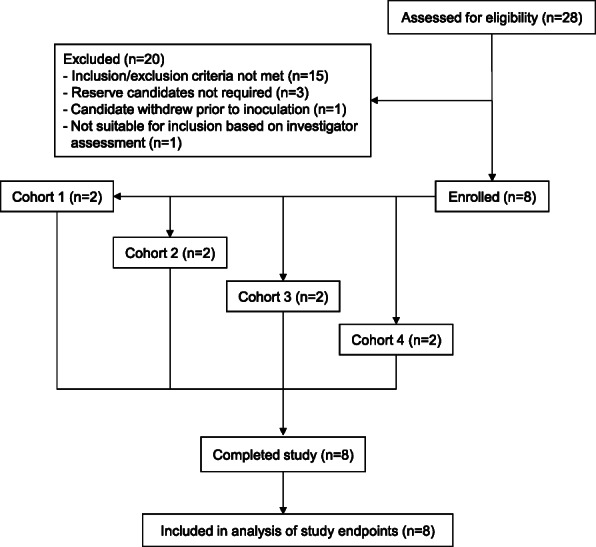
Trial profile. Volunteers meeting the eligibility criteria were enrolled in one of four GAP vaccine dose cohorts. Dose escalation was performed in cohorts 1 to 3; cohort 4 received the same dose as cohort 3

The study was conducted from 6 July 2017 to 11 September 2019. A total of 8 subjects were enrolled over four dose cohorts (Fig. 1). Subjects were healthy malaria-naïve males between the ages of 18 and 48 years; five subjects self-selected their race as White, two as Latin Hispanic, and one as Australian Aboriginal (Table 1). All subjects completed the study and were included in the analysis of study endpoints.

**Table 1:** Demographic profile of subjects

	Cohort 1		Cohort 2		Cohort 3		Cohort 4	
	Subject 1	Subject 2	Subject 3	Subject 4	Subject 5	Subject 6	Subject 7	Subject 8
GAP vaccine dose (viable parasites)	1800		1.8×10^5^		3×10^6^		3×10^6^	
Age (years)	40	48	26	19	32	18	29	20
Sex	Male	Male	Male	Male	Male	Male	Male	Male
Race	Latin Hispanic	Latin Hispanic	White	Australian Aboriginal	White	White	White	White
Body mass index (kg/m^2^)	23.0	27.3	18.1	21.7	26.7	23.8	26.8	23.0

*BMI* body mass index

### GAP vaccine infectivity

**Fig. 2:**
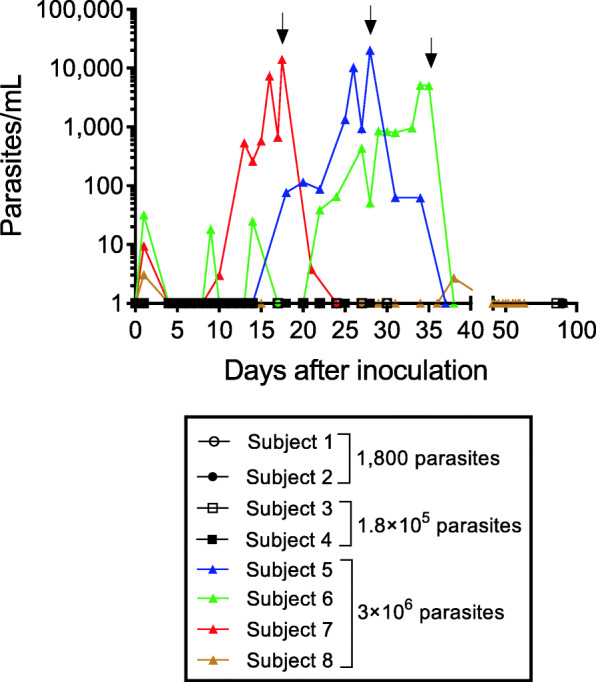
Infectivity of the GAP vaccine. Parasitemia was monitored by collecting blood samples and performing qPCR targeting the gene encoding *P. falciparum* 18S rRNA. Arrows indicate the commencement of treatment with artemether/lumefantrine for subjects who developed parasitemia. Time points at which parasitemia could not be detected were substituted with a value of 1 parasite/mL for the purpose of graphing on a logarithmic scale. Subject 7 was confirmed to be positive for influenza B at the time of antimalarial treatment

Parasitemia was not detected in subjects in cohort 1 (administered 1800 viable parasites) or cohort 2 (administered 1.8 × 10^5^ viable parasites) at any time-point during the study (Fig. 2). Parasitemia was detected in all four subjects in cohorts 3 and 4 (administered 3× 10^6^ parasites), although parasitemia was only detected very briefly and at a very low level (around the limit of detection) in one of these subjects (subject 8, day 1 and day 38, maximum 3 parasites/mL). The other three subjects developed significant parasitemia, the progression of which was highly variable between subjects. Antimalarial treatment with artemether/lumefantrine was initiated on day 17, day 28, and day 35 for these subjects at a parasitemia of 14,022 parasites/mL, 19,956 parasites/mL, and 5151 parasites/mL, respectively (Fig. 2, arrows). Parasitemia was rapidly cleared following artemether/lumefantrine treatment and no recrudescence was observed up to day 90 (Fig. 2).

### Safety

A total of 44 adverse events (AEs) were reported in this study, the majority of which were of mild severity (Table 2 and Additional File 1: Table S1). One AE met the protocol-defined criteria of a serious adverse event: a subject in cohort 4 experienced a joint dislocation on day 70 which required hospitalization and surgery. This event was not considered related to any study interventions. There were 15 AEs in 3 subjects (all administered the highest dose of 3 × 10^6^ viable parasites) that were considered to be possibly related to the GAP vaccine. Twelve of these AEs occurred in one subject (subject 7) and were mild symptoms/signs frequently associated with malaria (headache [2 events], myalgia, arthralgia [2 events], malaise, chills, pyrexia [2 events, maximum body temperature 38.9 °C], tachycardia [maximum heart rate 108 beats per minute], feeling hot, hyperhidrosis). However, this subject was confirmed by PCR to be positive for influenza B at the time of antimalarial treatment (which coincided with the onset of the majority of the signs/symptoms listed above); thus the symptoms and signs observed may not have been malaria-related. Another subject (subject 5) experienced mild fatigue (24 h duration) which was considered possibly related to the GAP vaccine.

**Table 2:** Summary of adverse events

	Cohort 1 (*N*=2)	Cohort 2 (*N*=2)	Cohort 3 (*N*=2)	Cohort 4^b^ (*N*=2)	Total (*N*=8)
Number of subjects with at least one adverse event [*n* (%)]; number of adverse events					
Any adverse event	2 (100%); 13	1 (50%); 3	2 (100%); 5	2 (100%); 23	7 (87.5%); 44
Adverse event of moderate intensity	1 (50%); 3	0 (0%); 0	1 (50%); 1	2 (100%); 3	4 (50%); 7
Adverse event of severe intensity	0 (0%); 0	0 (0%); 0	0 (0%); 0	1 (50%); 1^a^	1 (12.5%); 1^a^
Adverse event related to GAP vaccine	0 (0%); 0	0 (0%); 0	1 (50%); 1	2 (100%); 14	3 (37.5%); 15
Serious adverse event	0 (0%); 0	0 (0%); 0	0 (0%); 0	1 (50%); 1^a^	1 (12.5%); 1^a^

^a^The severe adverse event (joint dislocation) also met the criteria for a serious adverse event since it required hospitalization and surgery. This event was not considered related to the GAP vaccine. ^b^One subject in cohort 4 (subject 7) was confirmed to be positive for influenza B at the time of antimalarial treatment

Alloimmunization to minor erythrocyte antigens was observed in two subjects (subject 7 and subject 8) at the end of the study visit on day 90, which was considered related to the GAP vaccine. Subject 7 developed low titer anti-C and anti-P1 antibodies, while subject 8 developed low titer anti-c antibodies. Retrospective testing was performed on blood samples collected on day 21 (subject 7) and day 31 (subject 8). Subject 7 was positive for anti-P1 on day 21 (there was an insufficient sample to test for anti-C at this time point); subject 8 was negative for anti-c on day 31. The blood group and phenotype of these subjects, as well as the donor red blood cells used to manufacture the GAP master cell bank, are presented in Additional File 1: Table S2. Alloimmunization was not observed in any of the other 6 subjects administered the GAP vaccine.

### Immunogenicity

**Fig. 3:**
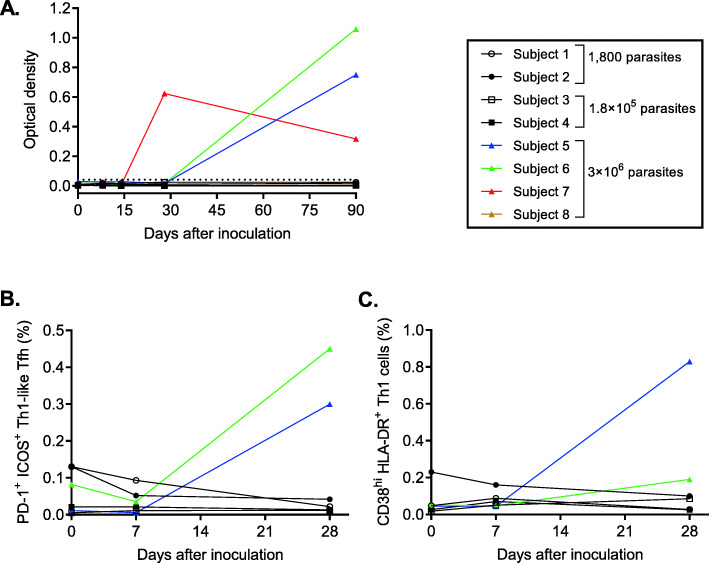
Immunogenicity of the GAP vaccine. The parasite-specific antibody response was determined by collecting blood samples and measuring anti-MSP2 IgG over the course of the study in each subject (**A**). The cell-mediated immune response was determined by measuring the frequency of activated (PD-1^+^ ICOS^+^) CD4^+^ Tfh cells (**B**) and activated (CD38^hi^ HLA-DR^+^) Th1 cells (**C**) in peripheral blood mononuclear cells over the course of the study in each subject. CD4^+^ T cell subsets were identified in B and C as indicated in Additional File 2: Figure S1. The cell-mediated immune response was not determined for cohort 4 (subject 7 and subject 8).

The three subjects who developed significant parasitemia following administration of 3 × 10^6^ parasites (subject 5, subject 6, and subject 7) also developed a malaria-specific antibody response. The antibody response to a key immunogenic blood-stage antigen (MSP2) was measured. The timing of the anti-MSP2 IgG response (Fig. 3A) and IgM response (Additional File 2: Figure S2) appeared to correlate with the development of parasitemia (Fig. 2). IgG and IgM antibody responses to whole parasite lysate were comparable to the MSP2 response in all subjects (Additional File 2: Figure S3). Furthermore, a malaria-specific cell-mediated immune response to the GAP vaccine was identified in two of the three subjects (subject 5 and subject 6; not tested in subject 7) administered 3× 10^6^ parasites, including increased expression of activation markers PD-1 and ICOS on Th1-like Tfh cells (Fig. 3B) and in the expression of activation markers CD38 and HLA-DR on Th1 cells (Fig. 3C) on day 28. There was no change in the frequencies of CD4^+^ T cells, Treg cells, Th1 cells, or Tfh cells in the peripheral blood of any of the subjects over the course of the study (Additional File 2: Figure S4).

### In vitro analyses of parasites rescued into culture

**Fig. 4:**
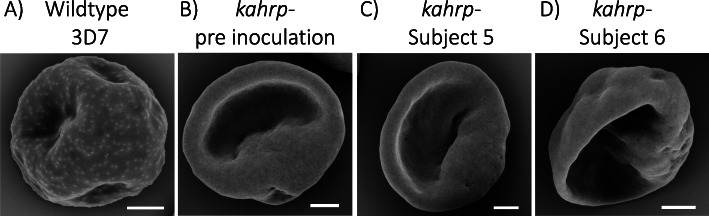
Scanning electron microscopy of parasite-infected erythrocytes. *P. falciparum* 3D7 wild-type (**A**), *P. falciparum* 3D7 *kahrp*– prior to inoculation (**B**), *P. falciparum* 3D7 *kahrp*– cultured ex vivo from subject 5 (**C**) and *P. falciparum* 3D7 *kahrp*– cultured ex vivo from subject 6 (**D**). Scale bar = 1 μm

**Fig. 5:**
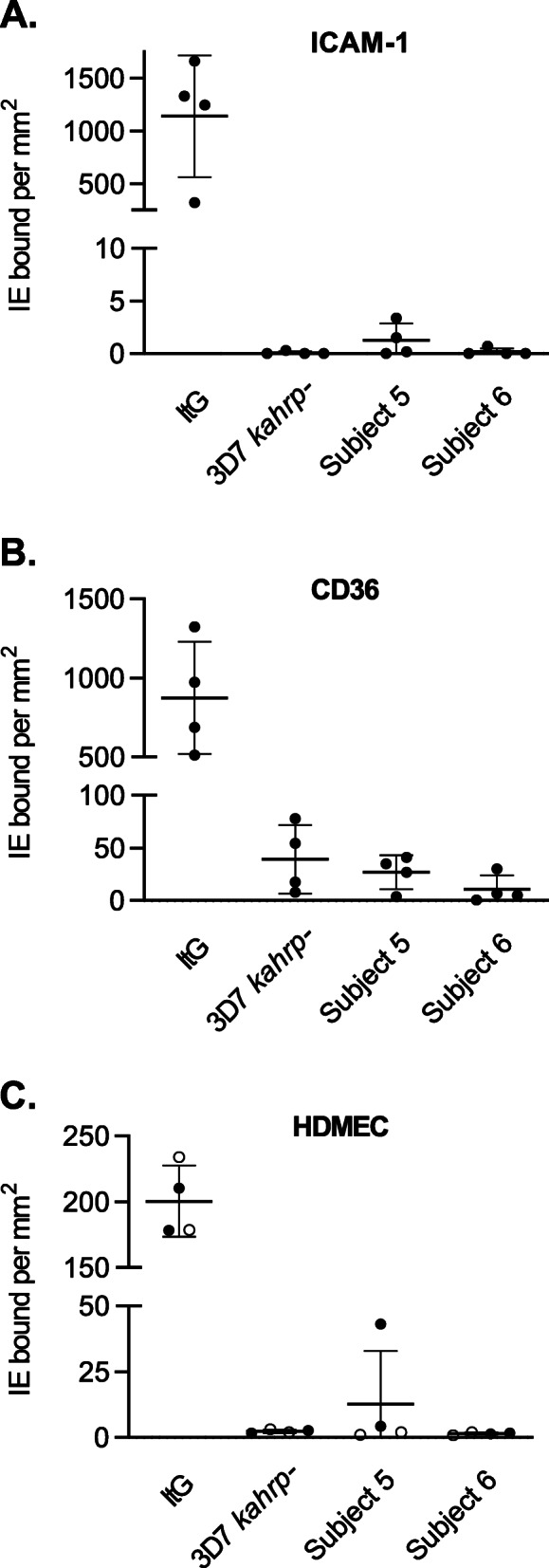
Cytoadherence of parasite-infected erythrocytes. The binding of infected erythrocytes to purified ICAM-1 (**A**) and CD36 (**B**) was determined under static conditions, and binding to unstimulated (○) or TNF-stimulated (●) human dermal microvascular endothelial cells (HDMEC) was determined under flow conditions (**C**). Assays were performed as described in Additional File 3: Text S2. Shown are the mean ± SD for 4 independent experiments. *P. falciparum* 3D7 *kahrp*– parasites cultured ex vivo from subject 5 and subject 6 were compared with parasites inoculated on day 0. There was no difference in cytoadherence characteristics when significance was determined by unpaired *t* test with Welch’s correction (*P* > 0.05). The laboratory isolate *P. falciparum* ItG was used as a positive control. IE: infected erythrocytes

**Fig. 6:**
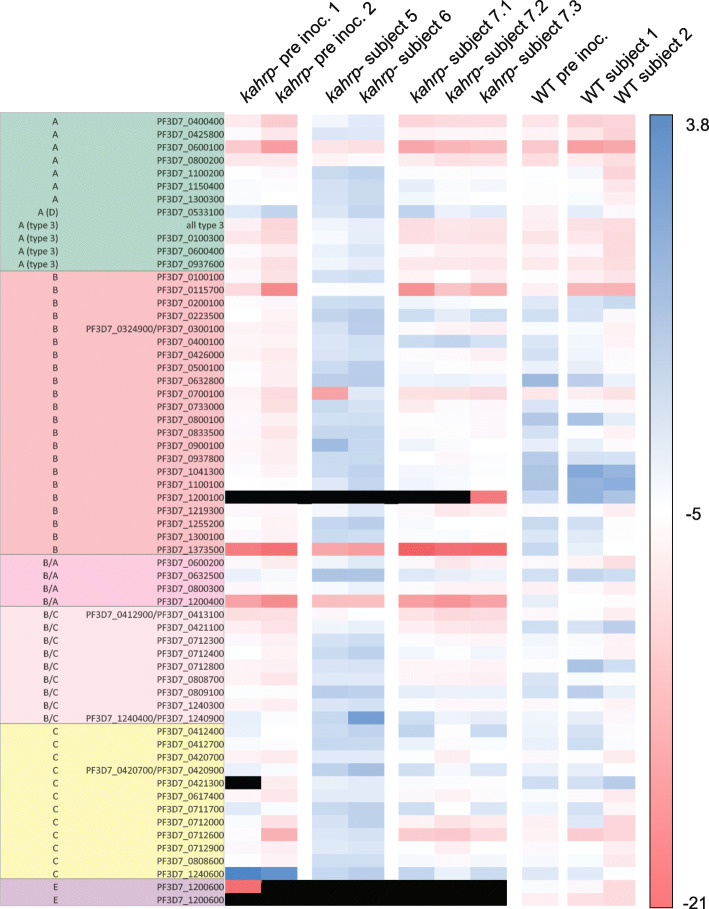
*Var* gene expression profiles. Log_2_ fold change in *var* transcript copy number relative to single copies in *P. falciparum* 3D7 gDNA. *P. falciparum* 3D7 *kahrp*– parasites cultured ex vivo from three subjects (*kahrp*– subject 5, *kahrp*– subject 6, and *kahrp*– subject 7) were compared with two replicates of parasites inoculated on day 0 (*kahrp*– pre inoc.1 and *kahrp*– pre inoc.2). Parasites were collected from subjects by taking blood samples immediately prior to antimalarial treatment initiation; three replicate samples were collected from subject 7 (*kahrp*– subject 7.1, *kahrp*– subject 7.2 and *kahrp*– subject 7.3). The duration of in vitro culture for each sample is specified in Additional File 3: Table S1. As a control, wild-type *P. falciparum* 3D7 parasites cultured ex vivo from two subjects (WT subject 1 and WT subject 2) in a controlled human malaria infection study using the induced blood stage malaria model [45] were compared with parasites inoculated on day 0 (WT pre inoc.). Blue bars indicate high expression, red bars indicate low expression, and black bars indicate that no expression was detected. Rows list *var* gene group and accession numbers

In vitro analyses of parasites rescued into culture from blood samples collected at peak parasitemia were performed to determine if any genotypic or phenotypic changes occurred during growth in vivo. Whole genome sequencing revealed that there were no significant genetic changes between the inoculated parasites and the ex vivo isolates (Additional File 3: Text S5), confirming that parasites had not reverted to a wild-type genotype, and had not acquired new mutations that may compensate for KAHRP deficiency. The ex vivo parasite-infected erythrocytes showed the same knob-minus appearance as infected erythrocytes inoculated on day 0 when examined using scanning electron microscopy (Fig. 4 and Additional File 3: Text S6) and retained an impaired cytoadherence phenotype in static and flow adhesion assays (Fig. 5 and Additional File 3: Text S7). Following growth in vivo, parasites switched away from primarily expressing a single group C *var* gene at baseline (inoculation), to elevated expression of most of the *var* repertoire in two subjects (subject 5 and subject 6) and a less broad but still fairly diverse pattern of expression in subject 7 (Fig. 6 and Additional File 3: Text S8). The most abundantly transcribed genes differed between the subjects and none had a published association with virulence (Additional File 3: Table S2) [44]. Wild-type *P. falciparum* 3D7 parasites from a CHMI study using the IBSM model [45] were analyzed as a control. The wild-type parasites used for inoculation of two subjects in that study expressed much of the *var* repertoire at elevated levels; the expressed repertoire following growth in vivo was largely unchanged in one subject but was less diverse in the other subject (Fig. 6).

## Discussion

This study represents the first clinical investigation of a genetically attenuated blood-stage human malaria vaccine. We demonstrate that *P. falciparum* 3D7 parasites lacking KAHRP are highly attenuated in vivo compared to their wild-type counterparts, with no parasitemia detectable up to 28 days following inoculation of up to 1.8× 10^5^ viable parasites. Inoculation of healthy subjects with 1800–2800 wild-type *P. falciparum* 3D7 parasites in CHMI studies using the IBSM model consistently results in detectable parasitemia within 5 days, and parasitemia typically reaches > 5000 parasites/mL 8 days after inoculation [21, 22, 25, 46, 47]. However, we found that *P. falciparum kahrp*– parasites were capable of generating a significant infection when administered at a high dose (3 × 10^6^ viable parasites).

The fact that *P. falciparum kahrp*– parasites are capable of generating a significant infection in humans was somewhat unexpected based on the well-characterized role of KAHRP in mediating the adherence of infected erythrocytes to the microvascular endothelium, which is an important process in promoting parasite survival by evading splenic clearance [18]. The in vivo growth observed in the current study appears to be consistent with the previously reported breakthrough infections (peak parasitemia of 0.02%) observed in 2 of 7 *Aotus* monkeys challenged with multiple doses of knobless *P. falciparum* parasites (up to 1× 10^7^ parasites) [17]. The delay in and variability of timing of onset of patent parasitemia in the subjects administered the highest dose suggests some in vivo adaptation of parasites to enable escape from host clearance mechanisms. In contrast to the highly reproducible growth pattern of parasites seen in experimental infection with unattenuated *P. falciparum* 3D7 parasites [48], there appears to be significant inter-individual variability in this process. Such variability would likely complicate any use of this approach for in vivo immunization. Because of the unexpected breakthrough in blood stage infection at the highest dose, we investigated for selection of phenotypic or genetic changes that had taken place to explain this observation. Genome sequencing of parasites rescued into culture at maximum parasitemia prior to antimalarial treatment confirmed that parasites had not reverted back to wild-type. Similarly, ex vivo cultured parasites retained their knob-minus phenotype, and exhibited impaired cytoadherence, particularly under flow conditions.

There was evidence that parasites switched their expression of *var* genes during in vivo growth. Parasites administered to subjects on day 0 expressed a single dominant group C *var* gene. This is consistent with previous evidence that, in the absence of phenotype selection, parasites often switch to expression of a group C *var* gene which appear to have a slower rate of silencing than other *var* gene types [49]. Following growth in vivo, parasites had switched away from the expression of the single group C *var* gene, suggesting selection in the naïve, human host, possibly for an adhesion phenotype. The increased diversity of *var* expression could also reflect the absence of selection for cytoadhesive variants, because the infected erythrocytes could not cytoadhere. If this were the case, then the altered environment within the host stimulated the de-repression of much of the *var* repertoire. Interestingly a similar observation of broad *var* de-repression has been made in CHMI studies using sporozoites [40]. In that case, passage through mosquitoes was inferred to have reset the epigenetic regulation of *var* genes, consistent with the broad, epigenetic reprogramming that occurs through sexual reproduction in higher order eukaryotes. The data we present suggests that the human host environment itself may stimulate *var* gene de-repression during the parasite’s asexual lifecycle.

Despite the well-characterized role of knobs in the cytoadherence and sequestration of parasite-infected erythrocytes, there is some evidence that erythrocytes infected with knob-minus parasites may be capable of binding weakly to the microvascular endothelium. Knobless *P. falciparum* clones were found to adhere to CD36 at a level comparable to the parent 3D7 strain under static conditions, but dramatic differences were observed under flow conditions [18]. Similar results were observed in the current study, with *P. falciparum kahrp*– parasites exhibiting some adhesion to CD36 under static conditions (albeit at considerably lower levels than the control strain) and almost no binding under flow conditions. These results are supported by recent research using engineered human capillaries which demonstrated that erythrocytes infected with knob-minus parasites can accumulate in the post-capillary space where shear rates are lower [50]. In contrast, knob-positive parasite-infected erythrocytes that were trypsinized to remove the surface presentation of PfEMP1 did not accumulate in vessels to any extent. Thus, we hypothesize that in the current study a limited number of *P. falciparum kahrp*– parasites were able to bind to the microvascular endothelium in areas where shear rates were low and avoid splenic clearance. This may have been facilitated by the switch in *var* gene expression observed during in vivo growth to present a range of PfEMP1s on the cell surface. An alternative hypothesis is that parasites may have accumulated in the spleen and were cleared by the immune system slowly enough to maintain an infection.

Immunogenicity results indicated that the GAP vaccine was capable of eliciting a malaria-specific antibody and cell-mediated immune response when administered at the highest dose (3 × 10^6^ viable parasites). Immunogenicity appeared to be dependent on parasite replication, evidenced by the fact that the subject administered the highest dose who did not develop parasitemia failed to generate a detectable antibody response. This finding may be consistent with evidence that killed whole parasite malaria vaccines require an adjuvant to induce robust immunity [51]. The anti-parasite humoral immune responses to the immunodominant blood-stage antigen MSP2 in this study were similar to those seen during natural infection against this and other blood-stage antigens, as well as when they were measured in CHMI studies using the IBSM model where subjects were infected with wild-type 3D7 parasites [52, 53]. As the preclinical primate data suggest a critical role of the spleen in the outcome of infection with knobless parasites, the effect of splenectomy on immunogenicity and clearance phenotype would require careful investigation in future clinical studies.

Although no serious safety concerns prevented completion of this stage of clinical development of the candidate vaccine, two considerations resulted in the vaccine not progressing to testing for protective efficacy in a challenge study with unattenuated parasites. Firstly, the fact that an immune response only developed in subjects who developed patent parasitemia some weeks after inoculation, and this parasitemia required termination by timed antimalarial chemotherapy, would impose significant practical limitations on the clinical development of this vaccine in the absence of other modifications to render the parasite avirulent. Secondly, two subjects administered the highest dose of GAP vaccine developed erythrocyte alloimmunization (anti-C and anti-P1 antibodies in one subject and anti-c antibodies in the other subject). This was unexpected based on our previous experience with CHMI studies using the IBSM model where alloimmunization attributed to the malaria challenge agent has not been reported previously (approximately 400 subjects in 32 studies). The fact that alloimmunization was observed in subjects who were administered a dose of parasites approximately 1000-fold higher than is typically administered in CHMI studies using the IBSM model involving wild-type *P. falciparum* 3D7 (3× 10^6^ viable parasites vs 2800 viable parasites) suggests that the dose of parasitized erythrocytes may be a factor. The total dose of erythrocytes (including non-parasitized) administered was similar to that routinely administered (approximately 1 × 10^8^ erythrocytes). We speculate that altered antigen presentation by parasitized erythrocytes with increased passage to the spleen may contribute to a higher probability of alloimmunization with the GAP vaccine. Of note, one of the 8 subjects administered a chemically attenuated blood-stage *P. falciparum* parasite vaccine in a pilot study also developed anti-c alloantibodies [10]. Although anti-P1 antibodies are not considered clinically important for transfusion reactions, anti-C and anti-c antibodies may result in a delayed transfusion reaction characterized by slow drop in hemoglobin over two weeks post-transfusion. Consultation with transfusion medicine specialists indicated there is no immediate risk if these subjects were to require emergency administration of unmatched Group O Rh (D) negative blood, and in the setting of routine blood transfusion a full cross-match would obviate such a reaction. Since both antibodies were of low titer, there is a possibility that they may diminish over time. Together, these two adverse safety findings precluded us continuing the study to test for protective immunity against virulent parasite challenge.

## Conclusions

Although the results of this study overall do not support the further clinical development of the *P. falciparum kahrp–* strain as a blood-stage GAP malaria vaccine, they do contribute valuable new information on the pathogenicity of *P. falciparum*. Additionally, this study acts as a proof-of-concept for the use of genetically modified blood-stage malaria parasites in humans. We have previously developed IBSM models for *P. falciparum* [23], artemisinin-resistant *P. falciparum* [54], *P. vivax* [55], and *P. malariae* [56]. The use of genetically modified parasites in CHMI studies using the IBSM model offers significant opportunities in the ongoing search for interventions to reduce the burden of malaria.

## Supplementary Information

**Additional File 1.** Supplementary methods and results for subject eligibility and safety assessments. **Text S1:** Subject eligibility criteria. **Table S1:** Incidence of adverse events by subject. **Table S2:** Red blood cell alloantibody test results for Cohort 4 and phenotype of donor red blood cells used for GAP master cell bank manufacture.

**Additional File 2.** Supplementary methods and results for immunogenicity assessments of GAP vaccine. **Text S1.** Methodology for determining antibody mediated immune response to GAP vaccine. **Text S2.** Methodology for determining cell mediated immune response to GAP vaccine. **Table S1.** Antibodies used for flow cytometry. **Figure S1.** The gating strategy used to identify CD4+ T cells and subsets, as well as the expression of T cell activation markers. **Figure S2.** The anti-MSP2 IgM response over the course of the study in each subject. **Figure S3.** The IgG and IgM response to whole parasite lysate over the course of the study in each subject. **Figure S4.** The frequency of CD4+ T cells, Treg cells, Th1 cells and Tfh cells in peripheral blood mononuclear cells over the course of the study in each subject.

**Additional File 3.** Supplementary methods and results for in vitro assessments of GAP vaccine. **Text S1.** Methodology for parasite genotyping. **Text S2.** Methodology for cytoadherence assays. **Text S3.** Methodology for scanning electron microscopy. **Text S4.** Methodology for var gene expression analysis. **Table S1.** Duration of in vitro culture of P. falciparum prior to harvest for RNA extraction for var gene transcription analysis. **Text S5.** Results of parasite genotyping. **Text S6.** Results of scanning electron microscopy analysis of parasite-infected erythrocytes. **Text S7.** Results of cytoadherence assays. **Text S8.** Results of var gene expression analysis. **Table S2.** Most abundantly expressed var genes.

## Data Availability

The datasets used and/or analyzed during the current study are available from the corresponding author on reasonable request.

## Abbreviations

AE: Adverse event
CHMI: Controlled human malaria infection
GAP: Genetically attenuated parasite
IBSM: Induced blood-stage malaria
KAHRP: Knob-associated histidine-rich protein
MSP2: Merozoite surface protein 2
PfEMP1: *Plasmodium falciparum* erythrocyte membrane protein 1
