# Supplementary material for: Safety, infectivity and immunogenicity of a genetically attenuated blood-stage malaria vaccine
Source: BMC Med. 2021 Nov 22;19:293. doi: 10.1186/s12916-021-02150-x (PMC8606250; doi:10.1186/s12916-021-02150-x)
Supplement: Supplementary file 1 — Additional File 1. Supplementary methods and results for subject eligibility and safety assessments. Text S1: Subject eligibility criteria. Table S1: Incidence of adverse events by subject. Table S2: Red blood cell alloantibody test results for Cohort 4 and phenotype of donor red blood cells used for GAP master cell bank manufacture. [file 12916_2021_2150_MOESM1_ESM.pdf]

**Additional File 1: Supplementary methods and results for subject eligibility and safety assessments**

## Text S1: Subject eligibility criteria

### Inclusion criteria:

Subjects who met all of the following criteria were included in the study:

Demography

I 01. Adult males between 18 and 55 years of age, inclusive who did not live alone (from Day 0 until at least the end of the anti-malarial drug treatment) and were contactable and available for the duration of the trial (maximum of 4 months).

I 02. Body weight, minimum 50.0 kg, body mass index (BMI) between 18.0 and 32.0 $kg/m^2$, inclusive.

I 03. Participants must have been willing and able to comply with scheduled visits, treatment plan, laboratory tests and other study procedures.

Health status

I 04. Confirmed as healthy by a comprehensive clinical assessment (detailed medical history and complete physical examination).

I 05. Normal vital signs after 5 minutes resting in supine position:

90 mmHg ≤ systolic blood pressure ≤ 140 mmHg,

50 mmHg ≤ diastolic blood pressure ≤ 90 mmHg,

40 bpm ≤ resting heart rate ≤ 100 bpm.

I 06. Normal standard 12-lead electrocardiogram (ECG) after 5 minutes resting in supine position; 120 ms<PR<210 ms, QRS<120 ms, QTcB<450 or QTcF≤450 ms with absence of second or third degree atrioventricular block or abnormal T wave morphology.

I 07. Laboratory parameters within the normal range, unless the Investigator considered an abnormality to be clinically irrelevant for healthy participants enrolled in this clinical investigation (for example, participants with asymptomatic mild hypercholesterolemia or glucose intolerance could be included). Serum creatinine, alkaline phosphatase, hepatic enzymes (aspartate aminotransferase, alanine aminotransferase), and total bilirubin (unless the participant had documented Gilbert syndrome) should not have exceeded the upper laboratory norm and haemoglobin must have been higher than the lower limit of the normal range.

I 09. Male volunteers could be included in the study if;

- their partner was of non-childbearing potential (post-menopausal or surgically sterile), or
- they agreed to use a double method of contraception: e.g. condom plus diaphragm or condom plus stable oral/transdermal/injectable hormonal contraceptive by female

partner from the first dose of GAP vaccine through the completion of the final anti-malarial treatment, or

- abstinent participants agreed to use a double barrier method if they commenced sexual relationships during the study and up to the last dose of the anti-malarial treatment.

Regulations

I 10. Having given written informed consent prior to undertaking any study-related procedure.

**Exclusion criteria:**

Subjects who met any of the following criteria were not included in the study:

Medical history and clinical status

E 01. Any history of malaria or participation in a previous malaria challenge study.

E 02. Must not have travelled to or lived (>2 weeks) in a malaria-endemic area/region during the past 12 months or planned travel during the study to a malaria-endemic region during the course of the study. In Australia areas to which travel was not permitted included coastal regions of Tropical North Queensland, the Northern Territory, and island regions of Northern Australia including the Torres Strait and any international destinations, countries or region within a country as listed by the Centres for Disease Control and Prevention (https://www.cdc.gov/malaria/travelers/country_table/a.html) where malaria infection is endemic, while they were infected with the GMO parasites and until they had been verified as parasite free (both asexual and gametocyte forms) and had completed the course of treatment.

E 03. Had evidence of increased cardiovascular disease risk (defined as >10%, 5-year risk for those greater than 35 years of age, as determined by the Australian Absolute Cardiovascular Disease Risk Calculator (Risk factors include sex, age, systolic blood pressure (mm/Hg), smoking status, total and HDL cholesterol (mmol/L) and reported diabetes status.

E 04. History of splenectomy.

E 05. Presence or history of drug hypersensitivity, or allergic disease diagnosed and treated by a physician or history of a severe allergic reaction, anaphylaxis or convulsions following any vaccination or infusion.

E 06. Presence of current or suspected serious chronic diseases such as cardiac or autoimmune disease (HIV or other immunodeficiency), insulin-dependent and NIDDM diabetes (excluding glucose intolerance if E03 is met), progressive neurological disease, severe malnutrition, acute or progressive hepatic disease, acute or progressive renal disease including microalbuminuria (albumin: creatinine >30mg/g), psoriasis, rheumatoid arthritis, asthma, epilepsy or obsessive compulsive disorder.

E 07. History of malignancy of any organ system (excluding non-spreadable skin cancers such as basal cell and squamous cell carcinoma or *in situ* cervical cancer), treated or untreated within 5 years of screening regardless of whether there is evidence of local recurrence or metastases.

E 08. Participants with schizophrenia, bi-polar disease, or other severe (disabling) chronic psychiatric diagnosis including history of depression or receiving psychiatric drugs or who had been hospitalized within the past 5 years prior to enrolment for psychiatric illness, history of suicide attempt or confinement for danger to self or others.

E 09. Known inherited genetic anomaly (known as cytogenetic disorders), e.g., Down's syndrome.

E 10. Presence of acute infectious disease or fever (e.g., sub-lingual temperature ≥ 38.5°C) within the five days prior to inoculation with malaria parasites.

E 11. Evidence of acute illness within the four weeks before trial prior to screening.

E 12. Significant inter-current disease of any type, in particular liver, renal, cardiac, pulmonary, neurologic, rheumatologic, or autoimmune disease by history, physical examination, and/or laboratory studies including urinalysis.

E 13. Participant had a clinically significant disease or any condition or disease that might affect drug absorption, distribution or excretion, e.g. gastrectomy, diarrhoea.

E 14. Participation and receipt of any investigational product within the 12 weeks preceding the study.

E 15. Participation in any research study involving blood sampling (more than 450 mL/ unit of blood), or blood donation to Red Cross (or other) blood bank during the 8 weeks preceding the reference drug dose in the study.

E 16. Participant unwilling to defer blood donations to the Australian Red Cross Blood Service (ARCBS), and organ donations during the study and for 6 months following the completion of the study.

E 17. Blood donation, any volume, within 1 month before inclusion.

E 18. Medical requirement for intravenous immunoglobulin or blood transfusions.

E 19. Participant who had ever received a blood transfusion.

E 20. Symptomatic postural hypotension at screening, irrespective of the decrease in blood pressure, or asymptomatic postural hypotension defined as a decrease in systolic blood pressure ≥20 mmHg within 2-3 minutes when changing from supine to standing position.

E 21. History or presence of alcohol abuse (alcohol consumption more than 40 g per day) or drug habituation, or any prior intravenous usage of an illicit substance.

E 22. Smoking more than 10 cigarettes or equivalent per day and unable to stop smoking during the study.

E 23. Ingestion of any poppy seeds within the 24 hours prior to the screening blood test (participants were advised by phone not to consume any poppy seeds in this time period).

E 24. Excessive consumption of beverages containing xanthine bases, including red bull, chocolate, etc. (eg, more than 400 mg of caffeine per day (more than 4 cups or glasses per day).

Interfering substance

E 25. Use of any prescription drugs, herbal supplements (including St John's Wort), over-the-counter (OTC) medication and/or dietary supplements (vitamins included) within 14 days prior to initial dosing until the conclusion of the study, except explicitly permitted by the investigator.

E 26. Any vaccination within the last 28 days.

E 27. Any recent (<6 weeks) or current systemic therapy with an antibiotic or drug with potential anti-malarial activity (chloroquine, piperaquine, benzodiazepine, flunarizine, fluoxetine, tetracycline, azithromycin, clindamycin, hydroxychloroquine, etc.).

General conditions

E 28. Any participant who, in the judgment of the Investigator, was likely to be noncompliant during the study, or unable to cooperate because of a language problem or poor mental development.

E 29. Any participant in the exclusion period of a previous study according to applicable regulations.

E 30. Any participant who could not be contacted in case of emergency for the duration of the trial and up to 2 weeks following end of study visit.

E 31. An employee of the sponsor or research site personnel directly affiliated with this study or their immediate family members defined as a spouse, parent, sibling, or child, whether biological or legally adopted.

E 32. Any participant without a good peripheral venous access.

Biological status

E 33. Positive result on any of the following tests: hepatitis B surface (HBsAg) antigen, anti-hepatitis B core antibodies (anti-HBcAb), anti-hepatitis C virus (anti-HCV) antibodies, anti-human immunodeficiency virus 1 and 2 antibodies (anti-HIV1 and anti HIV2 Ab).

E 34. Use of any drug excluded in the protocol unless there was an explanation acceptable to the medical investigator (e.g., the participant had stated in advance that they consumed a prescription or OTC product which contained the detected drug) and/or the Participant had a negative urine drug screen on retest by the pathology laboratory.

Specific to the study

E 35. Cardiac/QT risk:

- Known pre-existing prolongation of the QTcB/QTcF interval considered clinically significant,
- Family history of sudden death or of congenital prolongation of the QTc interval or known congenital prolongation of the QTc-interval or any clinical condition known to prolong the QTc interval. History of symptomatic cardiac arrhythmias or with clinically relevant bradycardia. Electrolyte disturbances, particularly hypokalaemia, hypocalcaemia or hypomagnesaemia,
- Electrocardiogram (ECG) abnormalities in the standard 12-lead ECG (at screening) which in the opinion of the Investigator was clinically relevant or would interfere with the ECG analysis,
- A history of clinically significant ECG abnormalities.

E 36. Known hypersensitivity to 4-aminoquinolines, artemether or other artemisinin derivatives, lumefantrine, or other arylaminoalcohols.

E 37. Unwillingness to abstain from consumption of quinine containing foods/beverages such as tonic water, lemon bitter, from inoculation (Day 0) to the end of the antimalarial treatment.

E 38. On dosing days:

- Ingestion of any drug since the recruitment interview (other than the doses administered in this study) that, in the opinion of the Investigator, could have compromised the study.
- Ingestion of any other drug, in the week prior to dosing or during the blood sampling period that, in the opinion of the Investigator, could have compromised the study, e.g.,

through pharmacokinetic or metabolic interactions, or analytical interference. However, the Investigator may have permitted the use of paracetamol or ibuprofen for the treatment of headache or other pain. If drug therapy other than paracetamol or drugs specified in the protocol was required during the study periods, a decision to continue or discontinue the participant’s participation was made by the Investigator based on the nature of the medication and the time the medication was taken.

- Failure to conform to the requirements of the protocol.
- Detection of any drug listed in the protocol in the urine drug screen unless there was an explanation acceptable to the medical investigator (e.g., the participant had stated in advance that they consumed a prescription or OTC product which contained the detected drug).
- Positive alcohol breath test.
- Vital signs outside the reference range and clinically significant.

## Table S1: Incidence of adverse events by subject

| Adverse Event Preferred Term | Number of events | | | | | | | |
|---|---|---|---|---|---|---|---|---|
| | Cohort 1 | | Cohort 2 | | Cohort 3 | | Cohort 4 | |
| | Subject 1 | Subject 2 | Subject 3 | Subject 4 | Subject 5 | Subject 6 | Subject 7 | Subject 8 |
| Abdominal discomfort | 0 | 1 | 0 | 0 | 0 | 0 | 0 | 0 |
| Anti-erythrocyte antibody positive | 0 | 0 | 0 | 0 | 0 | 0 | 1 | 1 |
| Arthralgia | 0 | 0 | 0 | 0 | 0 | 0 | 2 | 0 |
| Back pain | 0 | 0 | 0 | 0 | 0 | 0 | 0 | 1 |
| Chest discomfort | 0 | 1 | 0 | 0 | 0 | 0 | 0 | 0 |
| Chills | 0 | 0 | 0 | 0 | 0 | 0 | 1 | 0 |
| Cough | 0 | 0 | 0 | 1 | 0 | 0 | 1 | 1 |
| Diarrhoea | 0 | 1 | 0 | 0 | 0 | 0 | 0 | 0 |
| Dizziness | 1 | 0 | 0 | 0 | 0 | 0 | 0 | 0 |
| Dysphonia | 0 | 0 | 0 | 1 | 0 | 0 | 0 | 0 |
| Dyspnoea | 0 | 1 | 0 | 0 | 0 | 0 | 0 | 0 |
| Fatigue | 0 | 0 | 0 | 0 | 1 | 0 | 0 | 0 |
| Feeling hot | 0 | 0 | 0 | 0 | 0 | 1 | 1 | 0 |
| Headache | 0 | 3 | 0 | 0 | 0 | 0 | 2 | 0 |
| Hyperhidrosis | 0 | 0 | 0 | 0 | 0 | 0 | 1 | 0 |
| Influenza | 0 | 0 | 0 | 0 | 0 | 0 | 1 | 0 |
| Influenza like illness | 0 | 1 | 0 | 0 | 0 | 0 | 0 | 0 |
| Joint dislocation | 0 | 0 | 0 | 0 | 0 | 0 | 1 | 0 |
| Lymphocyte count decreased | 0 | 1 | 0 | 0 | 0 | 0 | 0 | 0 |
| Malaise | 0 | 0 | 0 | 0 | 0 | 0 | 1 | 0 |
| Musculoskeletal pain | 0 | 0 | 0 | 0 | 1 | 0 | 0 | 0 |
| Myalgia | 0 | 0 | 0 | 0 | 0 | 0 | 1 | 0 |
| Nausea | 0 | 1 | 0 | 0 | 0 | 0 | 0 | 0 |
| Nasal congestion | 0 | 0 | 0 | 0 | 0 | 0 | 1 | 0 |
| Oropharyngeal pain | 0 | 1 | 0 | 1 | 0 | 1 | 1 | 1 |
| Pyrexia | 0 | 0 | 0 | 0 | 0 | 0 | 2 | 0 |
| Rhinorrhea | 0 | 1 | 0 | 0 | 0 | 0 | 0 | 0 |
| Rhinovirus infection | 0 | 0 | 0 | 0 | 1 | 0 | 0 | 0 |
| Sinus congestion | 0 | 0 | 0 | 0 | 0 | 0 | 0 | 1 |
| Tachycardia | 0 | 0 | 0 | 0 | 0 | 0 | 1 | 0 |
| **TOTAL** | **1** | **12** | **0** | **3** | **3** | **2** | **18** | **5** |

**Table S2: Red blood cell alloantibody test results for Cohort 4 and phenotype of donor red blood cells used for GAP master cell bank manufacture**

| | **Subject 7 (male)** | **Subject 8 (male)** | **Donor Red Cells** |
|---|---|---|---|
| Blood Group | A Rh(D) +ve | O Rh(D) +ve | O Rh(D) -ve |
| Phenotype | D+ C- E+ c+ e-P1- | D+ C+ E- c- e+ | D- C+ E- c+ e+ K- Fya- Jka- M- N+ S+ |
| Red cells per dose | $1.6x10^8$ | $1.6x10^8$ | |
| Allo Ab screen pre-dose | Negative | Negative | |
| Allo Ab screen Day 21 | Anti-P1 | | |
| Allo Ab screen Day 31 | | Negative | |
| Allo Ab screen Day 90 | Anti C, Anti P1 (weak Ab titre) | Anti c (weak Ab titre) | |
