# Supplementary material for: Safety, infectivity and immunogenicity of a genetically attenuated blood-stage malaria vaccine
Source: BMC Med. 2021 Nov 22;19:293. doi: 10.1186/s12916-021-02150-x (PMC8606250; doi:10.1186/s12916-021-02150-x)

Additional File 2: Supplementary methods and results for immunogenicity assessments of GAP vaccine

Text S1: Methodology for determining antibody mediated immune response to GAP vaccine

P. falciparum 3D7 parasites were continuously cultured in human O+ red blood cells (provided by Australian Red Cross Lifeblood) in RPMI medium (Gibco) supplemented with 25 mM HEPES (Gibco), 30 µg/mL gentamicin (Gibco), 370 µM hypoxanthine (Sigma), 5% heat-inactivated human sera and 0.25% AlbuMAX II (Gibco). Parasite cultures were grown at 37°C in 1% O_2, 5% CO_2, 94% N_2 to schizont stage before enrichment in MACS® CS columns with custom magnetic separator. Purified parasite infected red blood cells were frozen in dry ice/ethanol mixture, then thawed rapidly at 37°C in a water bath. This freeze/thaw process was repeated for 5 cycles, followed by sonication in a chilled Bioruptor® Plus Sonication System (Diagenode). Lysed parasite material was centrifuged at 12,000 rpm and lysate supernatant was collected and stored at -80 °C.

Measuring the human antibody response to *P. falciparum* merozoite surface antigen 2 (MSP2) and to whole parasite lysate was performed using standard ELISA methods as previously described [27, 28]. Briefly, either recombinant MSP2 protein (0.5 µg/mL) or *P. falciparum* lysate (1.89×10^6 cells/mL equivalent) was coated onto 96-well Maxisorb plates (Nunc) and incubated overnight at 4°C. Plates were blocked with 5% skim milk for 2 hours at 37°C, before incubation with serum samples (1/100 for IgG testing and 1/250 for IgM testing) for a further 2 hours at room temperature. IgG antibody binding was detected using a goat polyclonal anti-human IgG HRP conjugate (1:4000; Invitrogen) for 1 hour at room temperature. IgM antibody binding was detected with mouse polyclonal anti-human IgM (1:1000; Invitrogen) for 1 hour, followed by goat polyclonal anti-mouse IgG HRP conjugate (1:1000; Invitrogen) for 1 hour. All washes were performed with PBS-Tween 0.05% and all antibodies were prepared in 0.5% skim milk. TMB Chromogen solution (Invitrogen) was added for 15 min at room temperature, and the reaction was stopped using 1M HCl. Antibody levels were measured as optical density at 450 nm using the Biotek Powerwave XS2 Microplate Reader.

Text S2: Methodology for determining cell mediated immune response to GAP vaccine

Peripheral blood mononuclear cells (PBMCs) were isolated as previously described [29, 30]. Flow cytometry on PBMCs was also performed as previously described [29]. Table S1 below shows antibodies purchased from either Biolegend (San Diego, CA) or BD Biosciences. Zombie Aqua Fixable Viability Kit (Biolegend) measured cell viability. The gating strategy used to identify $CD4^+$ T cells and subsets, as well as the expression of T cell activation markers, is illustrated in Figure S1 below.

Table S1: Antibodies used for flow cytometry

Marker	Colour	Clone
Ki67	BUV395	B56
CD45RA	BUV563	HI100
CD4	BUV737	OKT4
CXCR5	BV421	RF8B2
CCR4	BV605	1G1
CCR6	BV650	11A9
CD38	BV785	HIT2
PD-1	APC	EH12.1
CD25	AF700	2A3
HLA-DR	APC/Cy7	G46-6
CD3ε	FITC	UCHT1
CCR7	PerCP/Cy5.5	GO43H7
ICOS	PE	DX29
CXCR3	PE/CF594	1C6
CD127	PE/Cy7	HIL-7R-M21

Figure S1: The gating strategy used to identify CD4+ T cells and subsets, as well as the expression of T cell activation markers

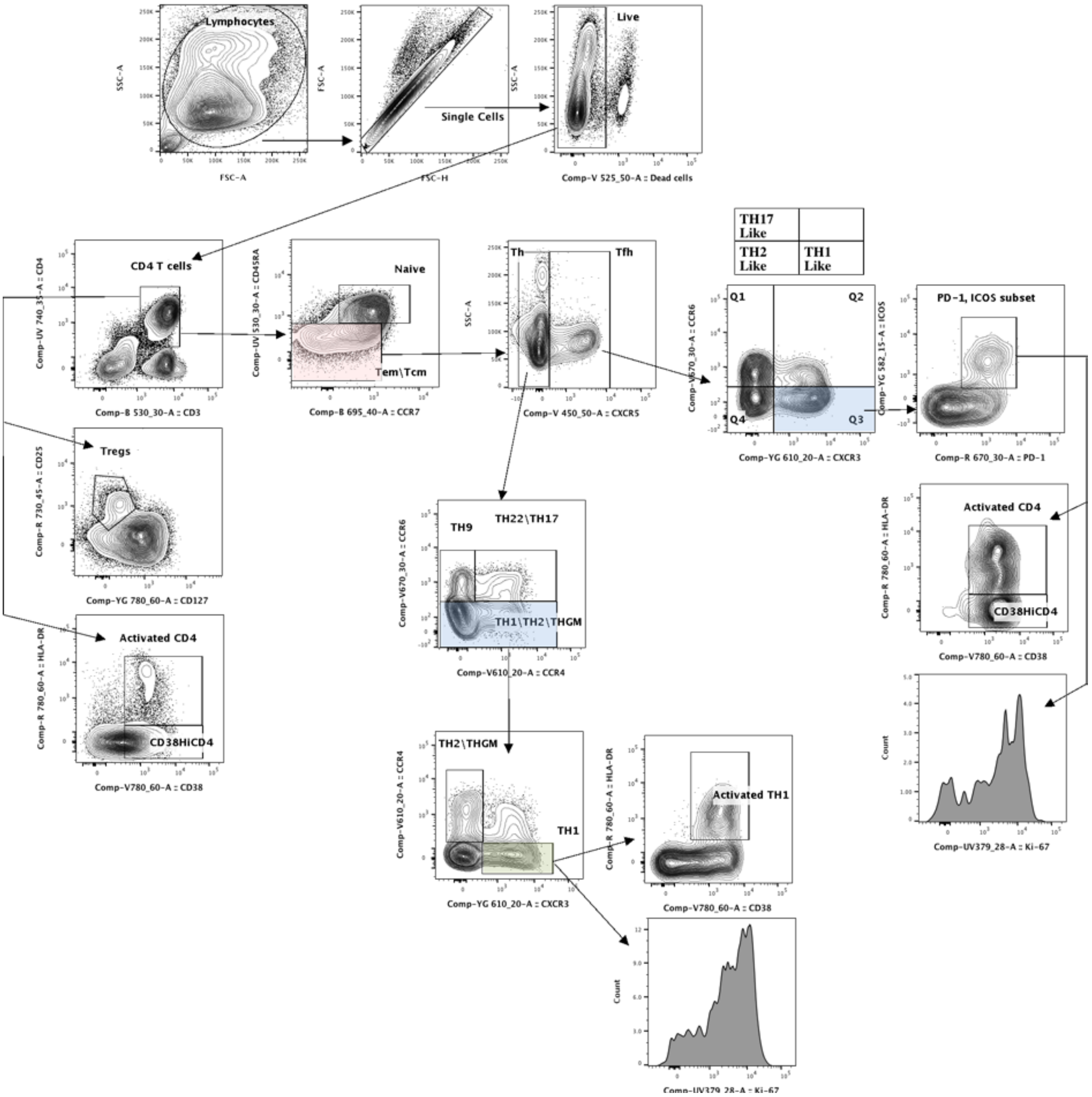

Figure S2: The anti-MSP2 IgM response over the course of the study in each subject

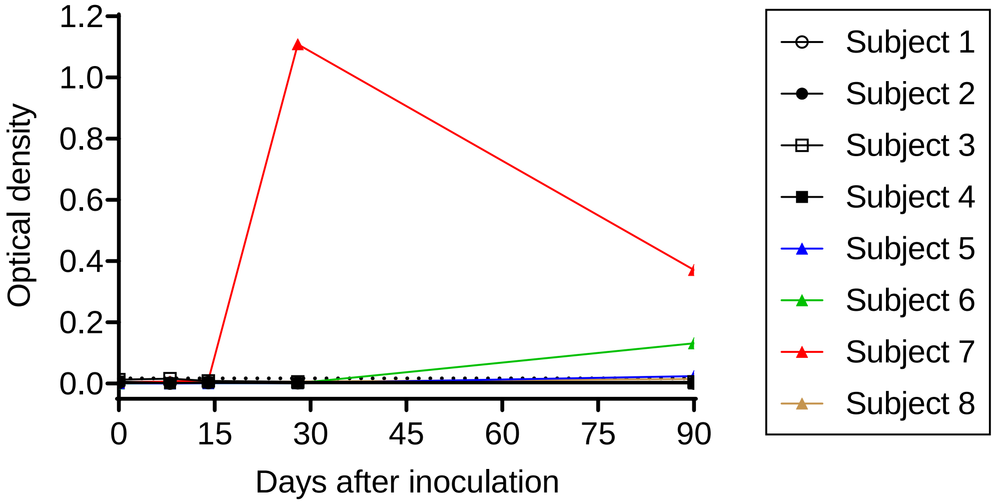

Figure S3: The IgG and IgM response to whole parasite lysate over the course of the study in each subject

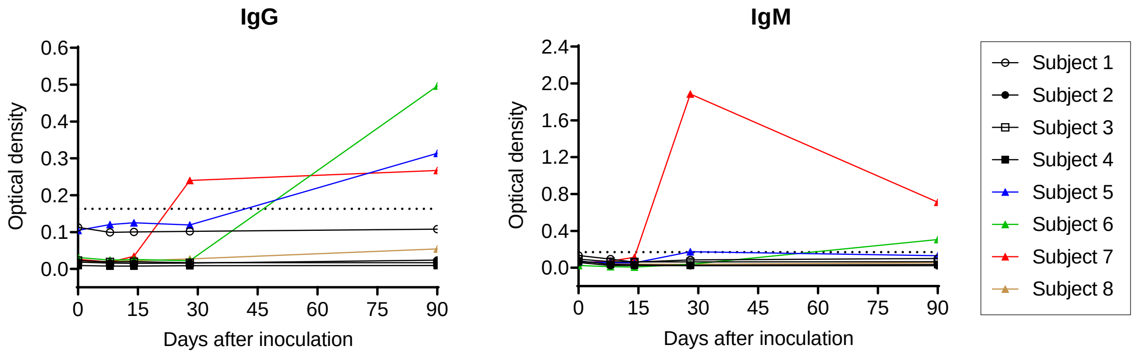

Figure S4: The frequency of CD4+ T cells, Treg cells, Th1 cells and Tfh cells in peripheral blood mononuclear cells over the course of the study in each subject

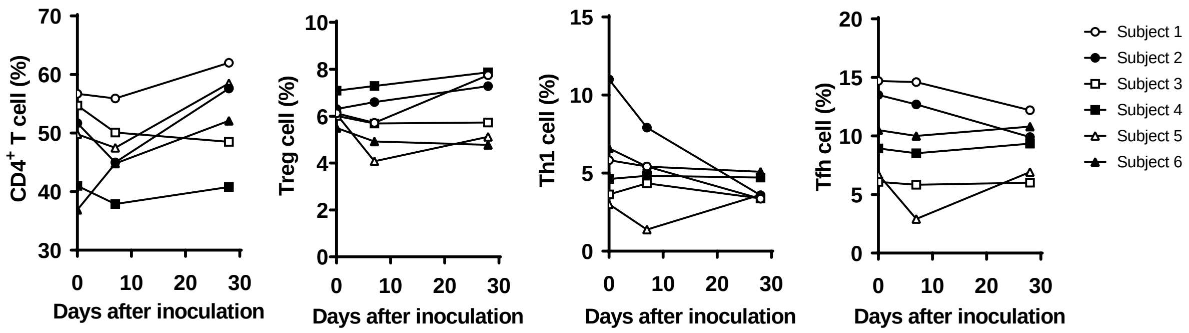

Supplement: Supplementary file 2 — Additional File 2. Supplementary methods and results for immunogenicity assessments of GAP vaccine. Text S1. Methodology for determining antibody mediated immune response to GAP vaccine. Text S2. Methodology for determining cell mediated immune response to GAP vaccine. Table S1. Antibodies used for flow cytometry. Figure S1. The gating strategy used to identify CD4+ T cells and subsets, as well as the expression of T cell activation markers. Figure S2. The anti-MSP2 IgM response over the course of the study in each subject. Figure S3. The IgG and IgM response to whole parasite lysate over the course of the study in each subject. Figure S4. The frequency of CD4+ T cells, Treg cells, Th1 cells and Tfh cells in peripheral blood mononuclear cells over the course of the study in each subject. [file 12916_2021_2150_MOESM2_ESM.pdf]
