# Supplementary material for: Safety, infectivity and immunogenicity of a genetically attenuated blood-stage malaria vaccine
Source: BMC Med. 2021 Nov 22;19:293. doi: 10.1186/s12916-021-02150-x (PMC8606250; doi:10.1186/s12916-021-02150-x)
Supplement: Supplementary file 3 — Additional File 3. Supplementary methods and results for in vitro assessments of GAP vaccine. Text S1. Methodology for parasite genotyping. Text S2. Methodology for cytoadherence assays. Text S3. Methodology for scanning electron microscopy. Text S4. Methodology for var gene expression analysis. Table S1. Duration of in vitro culture of P. falciparum prior to harvest for RNA extraction for var gene transcription analysis. Text S5. Results of parasite genotyping. Text S6. Results of scanning electron microscopy analysis of parasite-infected erythrocytes. Text S7. Results of cytoadherence assays. Text S8. Results of var gene expression analysis. Table S2. Most abundantly expressed var genes. [file 12916_2021_2150_MOESM3_ESM.pdf]

Additional File 3: Supplementary methods and results for in vitro assessments of GAP vaccine

Text S1: Methodology for parasite genotyping

Three DNA samples (subject 5, subject 6, and pre-inoculation control) were sequenced at Walter and Eliza Hall Institute on an Illumina NextSeq 500 instrument with parameters 2x81 cycles paired end plus a 6 base index read.

Sequences were aligned to reference PlasmoDB-29_Pfalciparum3D7 [31], using bowtie2 version 2.3.4.1, with parameters --sensitive-local --maxins 1000 [34]. Duplicate reads were removed using Picard tools MarkDuplicates version 2.9.4. The sequencing coverage was 120x to 276x depth.

Copy number analysis was done with the R package QDNAseq [35], version 1.14.0, with bin sizes of 5 kbp and 10kbp. The GRIDSS software package (v 2.2.2) [32] was used to find structural variants, which were screened to remove differences from the reference already present in the control sample.

Single Nucleotide Variants (SNVs) and small insertions and deletions were called with two different software tools, VarScan somatic version 2.3 [33], and SNVer version 0.5.3 [36]. Calls were filtered requiring minimum-read-depth = 10, alternate allele frequency in strain (AF) > 0.25, and discarding events near telomeres (defined as first and last 10% of each chromosome).

Text S2: Methodology for cytoadherence assays

Parasite culture

P. falciparum infected erythrocytes (IE) were cultured in RPMI 1640 with 30 mM HEPES, 11 mM glucose, 2 mM glutamine, 0.2% $NaHCO_3$, 25 mg/L gentamicin, 0.1 mM hypoxanthine and 10% human serum, pH 7.4. Isolated normal red blood cells were obtained from a commercial source. The laboratory isolate ItG (IT4var16) was used as a positive control [37].

Static adhesion assay

2.5 µL purified ICAM-1 and CD36 at 50 µg/mL and a PBS control were spotted in triplicate onto a 60 mm petri dish and incubated in a humidified container for 1 hour at 37°C. The protein was removed and the dish incubated for 1 hour in blocking solution (PBS/1% BSA) at 37°C. An IE suspension of 2% parasitaemia and 1.5% haematocrit in binding buffer (RPMI 1640 with 25 mM HEPES, 11 mM glucose, 2 mM glutamine, pH 7.2) was added and incubated at 37°C for 1 h with gentle resuspension every 10 minutes. Unbound IE were removed by washing with binding buffer and the bound IE fixed in 1% glutaraldehyde, stained with Giemsa, counted by microscopy and the mean IE per mm^2 surface calculated. The mean of the triplicates was calculated and non-specific binding to PBS subtracted to determine receptor specific binding in 4 independent experiments.

Flow adhesion assay

The assay was performed using primary Human Dermal Microvascular Endothelial Cells (HDMEC, Promocell, Germany) and the Cellix microfluidics system (https://www.wearecellix.com/) as previously described [38]. Briefly, HDMEC were cultured as per manufacturer's instructions and used up to passage 7. For the assay, cells were detached with Accutase® and seeded in Vena8 biochips (Cellix) coated with Attachment Factor. Medium was changed every hour and after 4-5 hours, when cells formed a confluent monolayer, an IE suspension of 2% parasitaemia and 5% haematocrit in binding buffer was flowed through at shear stress of 0.4 dyne/cm^2 for 5 minutes at 37°C. After a wash with binding buffer, the bound IE were counted in 15 fields by microscopy and the mean IE per mm^2 HDMEC surface calculated. Four independent experiments were performed and in two experiments HDMEC were stimulated overnight with 10 ng/mL TNF.

Text S3: Methodology for scanning electron microscopy

Parasite infected red blood cells were fixed with 0.05% glutaraldehyde for 25 minutes in PBS at room temperature and further in 2.5% glutaraldehyde for 1.5 hrs [39]. Fixed cells were washed in H_2O three times and subsequently adhered to poly-l-lysine coated glass coverslips for 5 minutes. Coverslips with bound cells were sequentially washed for 5 min each in 20%, 50%, 70%, 80%, 90%, 95% and 100% (×3) ethanol before transfer to a Lecia CPD300 critical point dryer, run for 24 fill cycles. Coverslips were gold-coated for 75 s at 25 mA using a Dynavac sputter coating instrument with rotating mount. The coating thickness was measured at ~0.3 nm on the internal quartz crystal microbalance. Images were acquired using an FEI Teneo SEM using the ETD detector in Optiplan mode, at a working distance of 5 mm, a beam current of 50 pA and a 2 kV accelerating voltage.

Text S4: Methodology for *var* gene expression analysis

Parasites were cultivated *in vitro* at 3% haematocrit for the duration specified in Table S1 below prior to harvesting infected erythrocytes from 60 mL of ring stage culture. Parasites were dissolved in 11 mL of trizol (Invitrogen) and RNA was extracted and purified using RNeasy mini columns (Qiagen) [43]. Contaminating DNA was digested with DNaseI (Qiagen) and digestion assessed by qPCR prior to another round of RNA purification (Qiagen RNeasy mini). RNA quality was assessed by Agilent bioAnalyser (RIN: 7.5-9.5). The harvested RNA was reverse transcribed and used to determine the *var* gene transcriptional profile by qRT-PCR using SYBR green master mix (Applied Biosystems) using published protocols [41] and primers [40]. Data were analysed as $2^{-(\Delta\Delta Ct)}$ using the skeleton binding protein 1 (SBP1) gene as the normalising control [42] and *P. falciparum* 3D7 gDNA as the calibrator.

Table S1: Duration of in vitro culture of *P. falciparum* prior to harvest for RNA extraction for *var* gene transcription analysis

**Parasite sample**	**Days in culture**
kahrp- pre inoc. 1	16
kahrp- pre inoc. 2	11
kahrp- subject 5	12
kahrp- subject 6	16
kahrp- subject 7.1	25
kahrp- subject 7.2	19
kahrp- subject 7.3	21
WT pre inoc.	24
WT subject 1	12
WT subject 2	6

Text S5: Results of parasite genotyping

No copy number differences were seen between the subject samples and the control sample. Structural variant (SV) analysis found the *kahrp* gene knockout in all three samples. The only new SV was a rearrangement in the sample from subject 5, affecting the first PfEMP1 *var* gene at the start of both chromosome 7 and chromosome 10. A small number of low-quality small variants (SNVs and indels) were observed, but no non-synonymous SNVs or indels in genes of known function.

Text S6: Results of scanning electron microscopy analysis of parasite-infected erythrocytes

Scanning electron microscopy (SEM) was performed to determine if breakthrough parasites had reverted to a wild-type knob phenotype. A reference image of wild-type 3D7 trophozoite-infected RBC is presented in Figure 4A showing abundant knobs on the external surface of the wild-type 3D7 parasite-infected RBCs. In contrast to this, no knobs are observed on the external surface of the *kahrp-* parasites prior to inoculation (Figure 4B). The *ex vivo* samples from subject 5 and 6 were imaged and show no knobs at the external surface (Figure 4 C,D). These results show that the breakthrough parasites imaged from subject 5 and 6 have not reverted to wild-type and present with the same knob-minus phenotype as the *kahrp-* parasite pre-inoculation.

Text S7. Results of cytoadherence assays

As shown in Figure 5, the cytoadherence characteristics of IE isolated from subject 5 and 6 are not significantly altered compared to 3D7 *kahrp*- IE. No binding to purified ICAM-1 (Figure 5A) and modest binding to purified CD36 (Figure 5B) could be detected under static binding conditions. There was no binding to HDMEC under flow conditions for 3D7 *kahrp*- IE and subject 6 IE, and the small amount of binding for subject 5 IE was not significantly different (Figure 5C). In contrast, the laboratory strain ItG did bind to the purified receptors and HDMEC as previously reported [37], indicating the robustness of the cytoadherence assays.

Text S8: Results of *var* gene expression analysis

The *var* genes most abundantly expressed by parasites from subject 5 and subject 6 had no published associations with virulence that could explain improved adhesion and thus escape of splenic clearance. In subject 5, a broad range of *var* genes were transcribed with a group B and a group B/A *var* genes dominating (Table S2 below). The gene PF3D7_0632500 in subject 5 might bind the endothelial cell receptor ICAM 1 via its DBLβ5 domain. This class of domains have been implicated in adhesion to ICAM 1 and cerebral malaria however this specific domain has not been shown to bind ICAM 1.

The most abundant *var* transcripts in parasites from subject 6 were detected by a single set of primers that cannot discriminate between *var* genes PF3D7_1240400 and PF3D7_1240900 (Table S2 below). These two *var* genes and one of the two most abundant *var* transcripts in subject 7 all bind the broadly expressed host receptor CD36. Adhesion to CD36 is not associated with severe malaria. The PfEMP1 PF3D7_1240600 expressed by the *kahrp-* parasites used for inoculation also binds CD36, thus the selection of parasites expressing PF3D7_1240400 and/or PF3D7_1240900 in subject 6 was probably not due to selection for adhesion to CD36. Whether the other domains of these PfEMP1s mediate adhesion to other receptors is unknown but could explain the observed selection of a switched parasite population in subject 6.

The level of expression of each gene for the three replicate *kahrp-* subject 7 samples were averaged and these values grouped with the *kahrp-* subject 5 and 6 gene expression values as replicates for comparison with the two *kahrp-* pre inoculation samples as replicates in a multiple t test comparison using the Holm-Sidak method to determine statistical significance in multiple comparisons with alpha=0.05. The expression of the single group C *var* gene PF3D7_1240600 was significantly reduced in the subjects' samples compared to the pre inoculation samples ($p<0.0001$). The expression of none of the other *var* genes differed significantly between the subjects' samples and the pre-inoculation samples. This was unsurprising given the variation between the individual *var* gene expression patterns between the subjects' samples, although notably subjects 5 and 6 were much more similar to each other than subject 7. If only subjects 5 and 6 were used as replicates to compare to the pre-inoculation controls then the *var* genes PF3D7_1240400/PF3D7_1240900 were significantly upregulated in the subjects' samples ($p=0.00018$) and PF3D7_1240600 was significantly down-regulated ($p<0.0001$).

Table S2: Most abundantly expressed *var* genes

**Parasite sample**	***Var* group**	***var* accession**	**Domain subtype and order**	**CD36 binding [44]**
kahrp- subject 5	B	PF3D7_0900100	NTSB3-DBLα0.16-CIDRα3.4-DBLδ1-CIDRγ12-ATSB6	nd
kahrp- subject 5	B/A	PF3D7_0632500	NTSB3-DBLα0.6-CIDRα3.2-DBLβ5-DBLγ13-DBLδ4-CIDRγ1-DBLε2-DBLε7-DBLε3-ATSB16	nd
kahrp- subject 6	B/C	PF3D7_1240400	NTSB3-DBLα0.16-CIDRα3.4-DBLδ1-CIDRβ1-ATSB7	binds
kahrp- subject 6	B/C	PF3D7_1240900	NTS-DBLα-CIDRα-DBLδ-CIDRβ-ATS	binds
kahrp- pre inoc.	C	PF3D7_1240600	NTSB3-DBLα0.20-CIDRα3.1-DBLδ1-CIDRβ1-ATSB23	binds
kahrp- subject 7	B	Pf3D7_0223500	DBLα0.16-CIDRα3.4-DBLδ1-CIDRβ1	nd
kahrp- subject 7	B	PF3D7_0400100	DBLα0.5-CIDRα2.10-DBLγ5-DBLδ1-CIDRβ1	binds

nd: not determined.
